# Discursive visibility of physical activity in school time reform: an analysis of district education bureaus’ official media narratives

**DOI:** 10.3389/fpubh.2026.1919160

**Published:** 2026-09-30

**Authors:** Xiting Wang, Fan Shi, Zhonghou Wang, Xiaoxiao Qu

**Affiliations:** School of Education Science, Qingdao University, Qingdao, China

**Keywords:** directed content analysis, discursive visibility, double reduction policy, physical activity, school time reform, social media

## Abstract

**Introduction:**

The organization of school time has been a focus of recent education reform, yet which school time reform practices appear in the official social media narratives of district education bureaus remains insufficiently understood.

**Methods:**

Taking Qingdao, China, as a case study, this study employed directed content analysis. A deductive coding framework of 3 first-level and 9 second-level nodes was first constructed from the literature and policy documents. This framework guided the retrieval of 376 articles from the official WeChat accounts of all 10 district education bureaus. Inductive analysis of these articles was then used to develop a typology of third-level reform narratives, which served as the final coding scheme.

**Results:**

The finalized typology comprised 25 third-level narrative types across instructional time, recess, and after-school services, with 704 occurrences coded in total. Each occurrence corresponds to one article in which a given node was identified, with each node recorded once per article. After-school services accounted for the largest share (60.1%), followed by recess (28.4%) and instructional time (11.5%). Physical activity-related practices were frequently reported across all three dimensions. Within instructional time, “Guaranteed PE instructional time” (30) and “PE instructional time quality assurance” (18) had the highest frequencies; in recess, “Ground space gamification” (38) was among the most frequently reported practices; within after-school services, “Curriculum-based club activities” (121) was the most frequently reported node, with sports clubs mentioned in 57.9% of these articles. The frequency of coded nodes also differed across districts.

**Discussion:**

A notable theme that emerged from the analysis was the frequent reporting of physical activity-related practices in official communication about school time reform. Within this theme, recess activities and after-school content appeared in more articles than structural changes to instructional time. Reporting was also selective: event-based and narrative-rich practices appeared more frequently, and physical activity was often described in relation to health and participation. The study contributes a transferable typology of reform narratives and demonstrates the value of social media data for examining public communication of school-based health practices.

## 1 Introduction

Globally, school time reform has become a major focus of education policy. How instructional hours, breaks, and extended activities are structured across the school day shapes the opportunities students have for learning, rest, and physical activity. Schools worldwide face a persistent temporal dilemma: how to allocate finite school hours to balance academic achievement with students’ physical, mental, and social development. A landmark report by the U. S. National Education Commission on Time and Learning, Prisoners of Time, argued that if time is used in the same way, extending it is pointless; to effectively improve educational quality, time must be used in new, different, and better ways (1). Decades later, this call for reimagining the temporal architecture of schooling remains urgent, particularly as schools are increasingly recognized as critical settings for health promotion.

China has emerged as a particularly instructive site for examining large-scale school time reform. In recent years, the country has issued a series of policy documents that bear on the restructuring of school time. The “Healthy China 2030” blueprint sets broad goals for integrating health into education (2); the 2022 National Compulsory Education Curriculum Standards allow schools to adjust class period durations and establish interdisciplinary thematic learning (3); and the “double reduction” policy directly targets the reorganization of instructional time, the extension and enrichment of recess, and the expansion of after-school services (4). Physical activity figures prominently within this policy landscape, reflected in mandates for no less than 1 h of daily physical activity at school (2), the State Council’s 2016 guidelines on strengthening school physical education (5), the 2022 revision of the Physical Education Law codifying the one-hour requirement into legal obligation (6), and the “Five-Education Integration” concept positioning physical development as an integral dimension of student growth (7). At the same time, this emphasis does not exhaust the policy agenda; it forms part of a broader effort that also addresses the reduction of homework burden, the integration of moral, intellectual, aesthetic, and labor education, and the overall quality of school life. Taken together, these policy developments point to a systematic effort to restructure school time for the benefit of student development.

These policy concerns intersect with several bodies of research on school time, each of which has examined how the allocation and organization of time within the school day have been studied and understood.

Research on instructional time, the most fundamental concept, initially focused on maximizing the efficiency of knowledge transmission within fixed class periods (8–10). Over time, the focus has shifted toward flexibility and structural optimization, driven by concerns for student physical and mental health. Studies have demonstrated that delaying school start times can improve adolescent sleep, health, and academic performance (11–14), highlighting how the temporal organization of the school day directly shapes health outcomes. New pedagogical approaches, such as project-based learning, have also prompted schools to break traditional class period boundaries through block scheduling or long-and-short class period systems (15–17), creating more flexible time blocks that can accommodate physical activity. International evidence from Germany (18–20), Japan (21), and France (22) further suggests that simply increasing or decreasing instructional time does not guarantee improved learning; rather, the structure and quality of time use, including time for physical activity, deserve equal attention.

Recess has been recognized as a critical temporal space for physical activity within the school day, and research in this area has addressed the link between time and student health. A recent systematic review confirms that recess brings multiple benefits to school-aged children, with physical health benefits being among the most consistently documented (23). Research has examined how embedding physical education content into recess can increase moderate-to-vigorous physical activity (MVPA) (24), how providing play equipment boosts activity levels (25), and how playground design shapes student activity patterns (26, 27), all demonstrating that the quality of recess time affects the quantity of physical activity students engage in. Incorporating traditional sporting games into recess also holds value for cultural identity and social inclusion (28). In China, the “double reduction” policy has created a supportive environment for extending recess and enriching its activity content, with many schools shifting from the traditional 10-min recess to a 15-min recess (29–31), thereby expanding the temporal window for school-based physical activity.

After-school services represent an extension of school time beyond the regular school day, and this strand of research has examined how extended hours can promote both physical health and holistic development. Systematic reviews show that after-school physical activity interventions can effectively improve cardiorespiratory fitness, muscle strength, and agility in children and adolescents (32–34), and that the quality of teacher-student interaction in these settings predicts students’ psychological need satisfaction and intrinsic motivation (35), illustrating how the use of extended time can produce both physical and motivational benefits. In China, the “double reduction” policy has positioned after-school services as a mechanism for promoting both holistic development and educational equity (36), though research also notes that schools in low-income communities may face disadvantages in accessing quality resources (37).

Despite this rich body of work, instructional time, recess, and after-school services are largely studied in isolation rather than as interconnected parts of how a school day actually unfolds for students. In China’s policy discourse, by contrast, these three dimensions are often discussed together, as they tend to change in tandem and influence one another. Examining them as an integrated system can therefore yield a more complete picture of how school time reform is unfolding.

One way to pursue such an integrated examination is through official media coverage, which offers a window into what reform practices are publicly narrated. Existing research has predominantly relied on policy document analysis, surveys, or case studies, while official social media accounts offer a complementary pathway. In China’s education governance system, district education bureaus operate as intermediaries between macro-level policy directives and micro-level school practices. Their official social media accounts have become a primary platform for publicly constructing and broadcasting an image of reform (38–40). Drawing on the policy-as-discourse perspective, which holds that official texts actively construct a version of reality rather than simply reflect it (41, 42), we treat these articles as a form of policy enactment discourse (43). Analyzing these articles therefore makes it possible to examine not only what reforms are communicated, but which reforms appear more frequently within the official communication of regional education governance.

This study uses a case of compulsory education schools in Qingdao, a sub-provincial city in Shandong Province. Drawing on a directed content analysis of WeChat articles from all 10 district education bureaus, the study first mapped the narrative types of school time reform practices appearing in official social media communication and then coded the number of articles in which each type appeared across the corpus. As physical activity-related types emerged as frequently reported, the analysis focused on their distribution across instructional time, recess, and after-school services. The central research question guiding this study is: What narrative types of school time reform practices appear in official social media narratives, and in particular, how do physical activity-related types feature within this broader landscape?

## 2 Materials and methods

### 2.1 Research design

This study employed a directed content analysis approach (44). Following Hsieh and Shannon’s framework, we combined deductive and inductive strategies. A preliminary coding framework was first developed deductively from the literature and policy documents. This framework guided the collection of a targeted textual corpus. Inductive analysis of this corpus was then used to generate a detailed typology of narrative types. After the typology was established, it was systematically applied back to the corpus for frequency coding. In this study, discursive visibility was defined as article-level frequency, meaning the number of articles in which a given narrative type was identified, rather than textual emphasis or reporting depth. The frequency patterns then allowed the study to identify which themes were most frequently represented across the corpus, and those themes were examined more closely. This second stage was not designed to test a prespecified hypothesis; rather, it followed patterns that emerged from the data themselves. In the present study, this process drew attention to physical activity as a recurring concern. This phased design allowed the study to move from a broad conceptual framework to a systematic mapping of school time reform narratives, and then to a closer analysis of the themes that appeared more frequently in official communication.

### 2.2 Case selection and analytical framework

This study selected Qingdao, China, as the case site. Qingdao is a sub-provincial city in Shandong Province, a prominent coastal open city, and a designated pilot area for basic education reform. It administers 10 districts and county-level cities, covering a total area of approximately 11,293 square kilometers with a resident population of about 10.52 million in 2025. The 10 districts differ in geographic size, population density, and level of urbanization. Reflecting these structural differences, they can be grouped into highly urbanized core districts (Shinan, Shibei, Licang, and Laoshan), newly developed districts (Chengyang and Huangdao), and outer county-level areas (Jimo, Jiaozhou, Pingdu, and Laixi). The four core districts cover approximately 5.2% of Qingdao’s total land area but account for about 27.6% of its resident population, while the four outer county-level areas cover about 70.8% of the land area and account for 41.0% of the population. These groups also differ in the size of their compulsory education school systems. The core districts contained 220 compulsory education schools, representing about 23.4% of the city total, whereas the outer areas contained 493 schools, representing 52.3%. School counts were obtained from district education statistics for 2024/2025. Available data also indicate that outer areas served a larger student population than the core districts, with approximately 430,000 students compared with about 270,000.

Since the introduction of the “double reduction” policy in 2021, Qingdao has moved earlier and gone further than many other cities in restructuring school time. Notable examples include Licang District’s pilot extension of standard recess from 10 to 15 min, and various districts’ experiments with flexible class scheduling. These practices respond directly to the policy goals of the Five-Education Integration concept, making Qingdao a well-suited case for study. The study focused on compulsory education schools (elementary and junior secondary) within these 10 districts.

The initial analytical framework for this study was constructed deductively by the first author, based on a review of the scholarly literature and an examination of national, provincial, and municipal policy documents (Table 1). This resulted in a framework comprising three first-level nodes (Instructional time, Recess, and After-school services) and nine second-level nodes (Table 2). The second-level nodes were grounded in both academic discourse and policy directives. For instance, under the instructional time dimension, “instructional time mandatory norms” and “instructional time structural optimization” were drawn from the literature on time allocation and flexibility, while “instructional time implementation monitoring” was informed by policy mandates concerning quality assurance. Although the policy review indicated that many school time reform requirements intersected with physical activity, the initial framework remained organized around the temporal structure of the school day rather than around any particular content area. This deductive framework defined the search domains and search terms. The final search terms are provided in Supplementary material 1. Initial search terms were derived from the first-level and second-level nodes of the coding framework. Supplementary terms emerged during iterative reading and were not assigned to specific nodes; they served only to broaden the retrieval net. The searches were conducted using the original Chinese terms. In Supplementary material 1, these Chinese terms are listed with English translations in parentheses.

**Table 1:** Policy basis for the coding framework of school time reform.

No.	Policy document	Issuing body	Year	Key provisions
P1	“Healthy China 2030” Blueprint (2)	General Office of the CPC Central Committee & General Office of the State Council	2016	Integrate health education into the national education system; ensure students engage in no less than 1 h of daily physical activity at school.
P2	Guidelines on Strengthening School Physical Education to Promote Students’ Physical and Mental Health (5)	General Office of the State Council	2016	Ensure students exercise for 1 h daily; improve physical education curricula; strengthen extracurricular physical exercise.
P3	Physical Education Law of the People’s Republic of China (2022 Revision) (6)	National People’s Congress	2022	Schools must offer full physical education classes in accordance with national regulations; ensure students participate in no less than 1 h of daily physical exercise during school.
P4	Opinions on Further Reducing the Homework Burden and Off-Campus Training Burden of Students in Compulsory Education (4)	General Office of the CPC Central Committee and General Office of the State Council	2021	Reduce total homework amount and duration; written homework for grades 3–6 ≤ 60 min, for grades 7–9 ≤ 90 min.
P5	Notice of the General Office of the Ministry of Education on Further Strengthening the Management of Physical Health of Primary and Secondary School Students (50)	Ministry of Education	2021	Ensure students go outside for moderate activities and relaxation during each recess; fully implement the long recess physical activity system with 30 min daily.
P6	Compulsory Education Curriculum Standards (2022 Edition) (3)	Ministry of Education	2022	Allow schools to adjust class period durations; establish interdisciplinary thematic learning activities.
P7	Opinions on Implementing the Student Physical Fitness Enhancement Plan (51)	Ministry of Education	2025	Require daily comprehensive physical activity for primary and secondary students ≥2 h; encourage two long recess sessions of ≥30 min (morning and afternoon).
P8	2025 Key Points for Citywide Education Work in Qingdao (52)	Qingdao Municipal Education Bureau	2025	Implement student physical fitness enhancement actions; ensure daily comprehensive physical activity ≥2 h; actively build an “Integration of Five-Domain in Education” education system.
P9	Qingdao Basic Education Expansion and Quality Improvement Three-Year Action Plan (2025–2027) (53)	Qingdao Municipal Education Bureau	2025	Promote the implementation of 15-min recess; create a rich and diverse after-school service course supermarket.
P10	Licang District Three-Year Action Plan for Primary and Secondary Education and Teaching Reform (2024–2026) (54)	Licang District Education and Sports Bureau	2023	Implement 15-min recess in all district primary schools; establish a phased promotion mechanism.

**Table 2:** Coding framework for school time reform (first-level and second-level nodes).

First-level node	Second-level nodes
Instructional time	Instructional time mandatory norms
	Instructional time structural optimization
	Instructional time implementation monitoring
Recess	Recess duration adjustment
	Recess activity enrichment
	Recess organization and management
After-school services	After-school service time structure
	After-school service content and programming
	After-school service management mechanisms

### 2.3 Data collection and corpus

The primary data source for this study was the official WeChat accounts of Qingdao’s 10 district-level education bureaus. WeChat is China’s most widely used social media platform; its official account function allows institutions to push text, images, and video directly to subscribers, making it a primary channel through which district bureaus publicly construct and broadcast an image of reform. The legitimacy of WeChat official accounts as a data source for investigating Chinese institutional practices is increasingly recognized in international scholarship (38–40). Each of the 10 district education bureaus maintained one official WeChat account, which was used to publicize education policies, school activities, and reform achievements and was equipped with a keyword search function. No formal communication mandate requires district education bureaus to report specific reform themes. These accounts were typically updated once per day, with occasional days featuring approximately two or three posts. Most posts were brief updates on school activities and policy implementation rather than lengthy reports. Although precise total posting volumes could not be reliably retrieved for the entire study period, the accounts were regularly updated throughout the study period.

Data were collected over a period spanning from July 2021 to March 2026. Within each official account, searches were conducted using the account’s built-in search function, and retrieved items were then screened manually. Searches based on initial search terms were able to capture a substantial proportion of relevant articles. However, because these node names represent analytical constructs that may not always correspond to the everyday language used in WeChat articles, supplementary terms were added to broaden the retrieval net and reduce the likelihood of missing relevant material. These supplementary terms were not specified in advance but emerged from iterative reading of the articles themselves as certain practice-oriented expressions recurred frequently. The iterative search continued until no substantially new practices were identified, at which point the search process was considered to have reached saturation. In total, 463 articles were retrieved across the 10 WeChat accounts.

The first author then manually screened these articles using three inclusion criteria. First, the content must be directly and substantially related to school time reform. Second, the article must contain concrete descriptions of specific practices or initiatives, rather than merely reposting policy announcements. Third, where multiple articles described highly similar activities, all were included. After applying the inclusion criteria, 87 articles were excluded, primarily because they merely reposted policy announcements without providing concrete descriptions of specific practices, or because their content was not substantially related to school time reform. The remaining 376 articles formed the final corpus for analysis. A flow diagram summarizing this screening process is provided in Figure 1. The regional distribution of this corpus is shown in Table 3.

**Figure 1:**
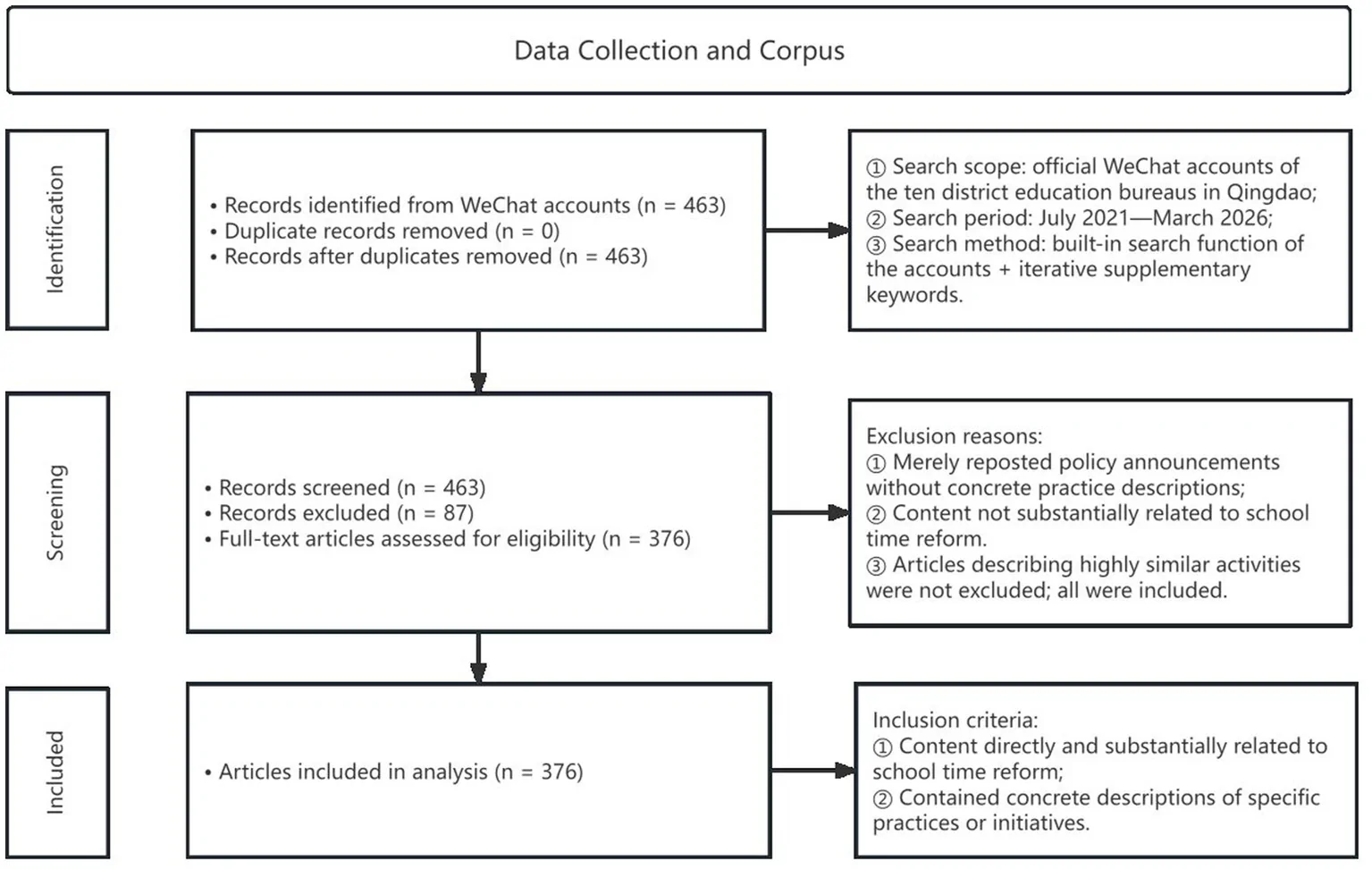
Flowchart of the data collection and screening process.

**Table 3:** Regional distribution of sample sources (*N* = 376).

District/city	Number of analyzed articles	Percentage (%)
Shinan District	62	16.6%
Licang District	56	14.9%
Shibei District	52	13.8%
Huangdao District	42	11.2%
Pingdu City	41	10.9%
Laoshan District	37	9.8%
Chengyang District	31	8.2%
Jiaozhou City	26	6.9%
Laixi City	15	4.0%
Jimo District	14	3.7%
Total	376	100%

### 2.4 Coding and analysis

The coding and analysis proceeded in three stages. All coding and analysis were conducted on the original Chinese text of the WeChat articles.

*Stage 1*: Inductive development of the coding instrument. The first author systematically reviewed all 376 articles to identify and extract distinct reform narrative types. Following the principles of inductive category development in qualitative content analysis (44), these narratives were recorded, compared across the corpus, and grouped through constant comparison. The category system was refined over multiple rounds until a stable set of third-level nodes was achieved, each representing a distinct narrative type with clear definitional boundaries. The resulting typology, which formed the final coding instrument, is presented in section 3.1. The complete codebook is provided as Supplementary material 2. Node definitions and example quotations in the codebook, as well as quotations cited in the Results and Discussion, were translated from Chinese to English by the first author. The translation aimed to preserve the original meaning and discursive framing of the Chinese text as closely as possible, rather than to produce a strictly literal rendering. Table 4 provides an excerpt from the codebook, with one illustrative example from each of the three dimensions.

**Table 4:** Excerpt from the codebook: examples of third-level node coding definitions across the three dimensions.

First-level node	Second-level node	Third-level node	Node code	Definition	Example quotes (source ID)
Instructional Time	Instructional time mandatory norms	Guaranteed PE instructional time	I-1	Refers to official WeChat public account articles reporting that schools have implemented or increased the number of PE class hours per week to meet national or local policy requirements for daily physical activity.	All primary and secondary schools in Licang District actively implement the Opinions on Implementing the Student Physical Fitness Enhancement Plan, taking measures such as… adhering to one PE class per day… to ensure that students engage in at least 2 h of comprehensive physical activity daily (2–3)
Recess	Recess duration adjustment	Recess time extension	R-1	Refers to official WeChat public account articles reporting that schools have extended the standard recess from 10 to 15 min or longer.	Since piloting the recess reform in 2023, Qingdao Chongli Primary School has embraced the health-first philosophy, turning the 15-min recess into a vitality station for students’ physical fitness improvement through scientific design and tiered activities (2–35)
After-school services	After-school service content and programming	Curriculum-based club activities	A-4	Refers to official WeChat public account articles reporting that schools have systematically integrated club activities into the after-school curriculum, including both sports-related and non-sports clubs.	Qingdao Zaoshan Primary School offers over 30 clubs including drama, roller skating, chess, football, rugby, wind ensemble, and aerobics (2–7)

*Stage 2*: Reliability testing. A second coder, a graduate student in education, was provided with the full codebook containing definitions and examples for all third-level nodes. After becoming familiar with the coding rules, both coders independently coded a randomly selected 15% subsample of the articles (*n* = 56). Inter-coder reliability, assessed using Cohen’s Kappa coefficient, was 0.80 across all 25 nodes, indicating a high level of overall agreement. Because several third-level nodes occurred infrequently, node-specific Kappa estimates would have been less informative; all coding disagreements were resolved through discussion before frequency coding began.

*Stage 3*: Systematic frequency coding. The first author then applied the finalized coding scheme to the entire corpus of 376 articles. The unit of analysis was the individual article: the presence of a given node was recorded once per article, regardless of how many times it appeared within that article. Consequently, a brief mention and an extensive report were treated equivalently. As defined in section 2.1, discursive visibility refers to article-level frequency. References to visibility in the Results and Discussion refer to this measure unless otherwise stated. This two-pass approach, in which category development was followed by systematic frequency coding, ensured that all articles were evaluated against a stable and consistently defined set of categories. The resulting frequency distribution provides the basis for the quantitative analysis reported in section 3. A corpus index containing district/account names, article ID, article titles, publication dates, primary retrieval terms, and coded nodes is provided as Supplementary material 3.

Three methodological clarifications should be noted. First, all frequency counts are based exclusively on the 376 included articles, not on the total number of posts published by each account. The results therefore reflect the discursive visibility of the sampled narratives and should not be generalized to overall district communication activity or taken as evidence of actual school-level implementation. Second, not all 25 third-level nodes were identified in every included article; articles that did not contain any assignable node contributed zero to the cumulative frequency. Third, cross-coding was permitted when a single article addressed multiple dimensions of school time reform simultaneously. The cumulative frequency across all nodes is therefore best interpreted as an indicator of the discursive visibility accorded to different reform narratives within the included corpus, rather than as a direct measure of actual implementation.

Before presenting the results, a conceptual point is worth noting. The third-level nodes capture two complementary aspects of school time reform. The first concerns time quantity and structure, referring to adjustments to the duration and organization of school time segments. The second concerns time quality, referring to the substantive activities that fill these reconfigured temporal frames. The frequency analysis in section 3 thus reflects both dimensions, recognizing that restructuring time yields limited benefits unless accompanied by improvements in the quality of activities (10).

### 2.5 Ethical considerations

This study was conducted in accordance with established academic ethical principles. All data were collected exclusively from publicly accessible, officially verified WeChat official accounts and are intended for public dissemination. The study did not involve the collection of any private or personally identifiable information. Given the exclusive use of publicly available, non-sensitive institutional data, it was determined to be exempt from full institutional review board (IRB) review.

## 3 Results

### 3.1 Overview of third-level nodes

Through inductive analysis of the 376 articles, 25 third-level nodes were identified across three dimensions, with 7 nodes related to instructional time, 8 to recess, and 10 to after-school services (Table 5).

**Table 5:** Overview of third-level nodes in school time reform.

First-level node	Second-level node	Third-level node
Instructional time	Instructional time mandatory norms	Guaranteed PE instructional time
		Guaranteed labor education instructional time
		Guaranteed aesthetic education instructional time
	Instructional time structural optimization	Flexible class period scheduling
		Weekly block reorganization
	Instructional time implementation monitoring	Supervision and inspection arrangements
		PE instructional time quality assurance
Recess	Recess duration adjustment	Recess time extension
		Diversified recess time configurations
	Recess activity enrichment	Ground space gamification
		Educational indoor activities
		Ball-game recess activities
		Interdisciplinary extension of recess activities
	Recess organization and management	Phased promotion mechanism
		Safety assurance mechanism
After-school services	After-school service time structure	Segmented activity time model
		Flexible after-school service boundaries
	After-school service content and programming	Menu-based course provision
		Curriculum-based club activities
		After-school homework management
	After-school service management mechanisms	After-school service supervision
		Parental resource integration
		Community resource integration
		University resource integration
		Collaborative education mechanism

These nodes capture two complementary aspects of school time reform, namely time quantity and structure, and time quality. The distribution of these 25 nodes shows a gradient in the number of narrative types, which forms the basis for analyzing discursive visibility in the following sections. After-school services, with 10 nodes, has the largest number of narrative types, spanning time structure, content programming, and multi-stakeholder management. Recess reform, with 8 nodes, covers time adjustment and activity enrichment. Instructional time, with 7 nodes, centers on PE guarantees and structural optimization. Several nodes were specifically related to physical activity, including “Guaranteed PE instructional time,” “PE instructional time quality assurance,” “Ground space gamification,” “Ball-game recess activities,” and “Curriculum-based club activities”. These physical activity-related nodes appeared across all three dimensions.

### 3.2 Overall distribution of discursive visibility

Across the 376 articles, 704 occurrences of third-level nodes were identified. Table 6 presents their distribution by first-level dimension. After-school services accounted for the largest share (423 occurrences, 60.1%), followed by recess (200 occurrences, 28.4%) and instructional time (81 occurrences, 11.5%). In line with the definition in section 2.1, discursive visibility was measured as article-level frequency. The frequencies reported here therefore indicate how many articles mentioned a given practice, rather than how extensively or in how much detail it was discussed. The gradient, from 60.1% for after-school services to 11.5% for instructional time, indicates that discursive visibility was unevenly distributed across the three dimensions in official communication.

**Table 6:** Frequency distribution of school time reform by dimension.

First-level dimension	Frequency	Percentage
Instructional time	81	11.5%
Recess	200	28.4%
After-school services	423	60.1%
Total	704	100%

This study was initially designed to map the landscape of school time reform practices broadly, without privileging any particular content area. As coding progressed, however, physical activity-related narratives emerged as a frequently reported pattern across all three dimensions. This pattern was partly attributable to the nature of recess, which is inherently linked to physical activity. Yet physical activity also appeared frequently in instructional time and after-school services, dimensions that are not confined to physical education. That physical activity was frequently reported across all three dimensions suggests that official communication frequently associated school time reform with physical activity-related practices.

### 3.3 Instructional time: low visibility, concentrated on PE guarantees

Instructional time reform accounted for the smallest share of coded occurrences, with 81 occurrences across seven third-level nodes (Table 7).

**Table 7:** Frequency statistics for instructional time dimension.

Second-level node	Third-level node	Frequency
Instructional time mandatory norms	Guaranteed PE instructional time	30
	Guaranteed labor education instructional time	8
	Guaranteed aesthetic education instructional time	6
Instructional time structural optimization	Flexible class period scheduling	7
	Weekly block reorganization	6
Instructional time implementation monitoring	Supervision and inspection arrangements	6
	PE instructional time quality assurance	18

Instructional time had the lowest discursive visibility among the three dimensions. The instructional time narratives displayed two main characteristics. First, structural optimization narratives, specifically “Flexible class period scheduling” (7 occurrences) and “Weekly block reorganization” (6 occurrences), were infrequent. Reports of Qiyuan School’s “Integration-Innovation Week” and Chengyang District’s long-and-short class period reform were mentioned but infrequent. Second, “Supervision and inspection arrangements” (6 occurrences) appeared as a mechanism for ensuring compliance with time-related mandates.

Within this limited set of narratives, PE-related narratives were the most frequently reported. “Guaranteed PE instructional time” (30 occurrences) and “PE instructional time quality assurance” (18 occurrences) together accounted for 59.3% of all instructional time narratives. Chengyang District was featured for fully implementing daily PE classes. Shinan District promoted its “2 + 1 + 2” model (2 basic PE classes, 1 elective rotating class, and 2 physical activity classes). Changsha Road Primary School in Shibei District was reported for its “class-by-class training” approach, embedding a 10-min fitness segment into every PE lesson. Narratives around “Guaranteed labor education instructional time” (8 occurrences) and “Guaranteed aesthetic education instructional time” (6 occurrences) were, by contrast, rarely reported.

### 3.4 Recess: structural reorganization and activity enrichment

Recess reform occupied an intermediate position, with 200 occurrences across eight third-level nodes (Table 8).

**Table 8:** Frequency statistics for recess dimension third-level nodes.

Second-level node	Third-level node	Frequency
Recess duration adjustment	Recess time extension	24
	Diversified recess time configurations	92
Recess activity enrichment	Ground space gamification	38
	Educational indoor activities	19
	Ball-game recess activities	9
	Interdisciplinary extension of recess activities	7
Recess organization and management	Phased promotion mechanism	5
	Safety assurance mechanism	6

The recess reform narratives were characterized by more frequent reporting of structural reorganization than simple time extension. “Diversified recess time configurations” (92 occurrences) far exceeded “Recess time extension” (24 occurrences). The reported approaches included morning and afternoon long recess sessions alongside shorter breaks, as well as grade-differentiated recess schedules. Licang District’s pilot extension of recess to 15 min, launched in 2023, exemplifies the “Recess time extension” narrative, and broader structural reorganization was also reported across other districts. Recess organization and management were rarely reported: “Phased promotion mechanism” (5 occurrences) and “Safety assurance mechanism” (6 occurrences) were infrequently mentioned.

Physical activity-related practices were frequently reported in connection with recess enrichment. “Ground space gamification” (38 occurrences) was the most frequently reported activity-related narrative. The relevant articles described distinctive systems for designing and organizing ground-based games, and traditional games such as rubber band jumping, beanbag throwing, and eagle-catching-chicks were said to have returned to school life. “Ball-game recess activities” (9 occurrences), “Educational indoor activities” (19 occurrences), and “Interdisciplinary extension of recess activities” (7 occurrences) added further variety. The frequent reporting of ground space gamification and diversified recess configurations indicates that, in official narratives, the discursive visibility of recess reform centered on activity enrichment rather than simple time extension.

### 3.5 After-school services: physical activity in extended school time

After-school services were the most frequently narrated dimension, with 423 occurrences across 10 third-level nodes (Table 9).

**Table 9:** Frequency statistics for after-school services dimension.

Second-level node	Third-level node	Frequency
After-school service time structure	Segmented activity time model	62
	Flexible after-school service boundaries	22
After-school service content and programming	Menu-based course provision	47
	Curriculum-based club activities	121
	After-school homework management	78
After-school service management mechanisms	After-school service supervision	23
	Parental resource integration	30
	Community resource integration	13
	University resource integration	9
	Collaborative education mechanism	18

The relevant articles showed two main patterns in the reporting of time allocation and content. First, a “Segmented activity time model” (62 occurrences) was frequently described. For example, some articles reported a first segment dedicated to homework tutoring under teacher supervision and a second reserved for club activities and interest-based courses. “After-school homework management” (78 occurrences) reported tiered homework designs and “homework cut-off” mechanisms aimed at protecting student sleep. “Flexible after-school service boundaries” (22 occurrences) described staggered dismissal times. Second, “Menu-based course provision” (47 occurrences) was associated with an emphasis on personalized choice, exemplified by Shinan District’s digital after-school service platform. Among management mechanisms, “Parental resource integration” (30 occurrences) was the most frequently reported, followed by “After-school service supervision” (23 occurrences) and “Collaborative education mechanism” (18 occurrences). “Community resource integration” (13 occurrences) and “University resource integration” (9 occurrences) appeared less frequently.

Physical activity-related practices were frequently mentioned in the after-school service content. “Curriculum-based club activities” (121 occurrences) was the single most frequently reported narrative across all 25 nodes. Of the 121 articles with this node, 70 articles (57.9%) specifically mentioned at least one sports or physical activity club, while the remaining 51 articles referred to non-physical-activity clubs such as music, art, drama, and chess. Qingdao Zaoshan Primary School in Licang District, for instance, listed over 30 clubs including roller skating, football, rugby, drama, maker robots, and chess, with sports clubs forming a substantial portion.

### 3.6 Regional variation in discursive visibility

When the data were disaggregated by district (Table 10), discursive visibility also differed across districts.

**Table 10:** Distribution of third-level node occurrences across districts and dimensions.

District	No. of schools	Instructional time	Recess	After-school services	Total	Mean distinct nodes per included article	Node occurrences per school	Primary discursive emphasis
Shinan District	39	27(12.9%)	23(10.9%)	160(76.2%)	210	3.39	5.38	After-school-centered
Shibei District	89	13(10.7%)	19(15.7%)	89(73.6%)	121	2.33	1.36	After-school-centered
Laoshan District	38	4(6.1%)	10(15.4%)	51(78.5%)	65	1.76	1.71	After-school-centered
Pingdu City	162	5(12.5%)	7(17.5%)	28(70.0%)	40	0.98	0.25	After-school-centered
Licang District	54	14(10.4%)	69(51.1%)	52(38.5%)	135	2.41	2.50	Recess-centered
Huangdao District	146	7(20.0%)	17(48.6%)	11(31.4%)	35	0.83	0.24	Recess-centered
Chengyang District	83	3(9.4%)	17(53.1%)	12(37.5%)	32	1.03	0.39	Recess-centered
Jiaozhou City	87	4(12.5%)	21(65.6%)	7(21.9%)	32	1.23	0.37	Recess-centered
Jimo District	166	1(4.8%)	10(47.6%)	10(47.6%)	21	1.50	0.13	Low-activity
Laixi City	78	3(23.1%)	7(53.8%)	3(23.1%)	13	0.87	0.17	Low-activity
Total	942	81(11.5%)	200(28.4%)	423(60.1%)	704	–	–	–

In four districts, recess-related nodes accounted for the largest proportion of that district’s coded occurrences: Licang (51.1%), Huangdao (48.6%), Chengyang (53.1%), and Jiaozhou (65.6%). In another four districts, after-school service-related nodes accounted for the largest proportion: Shinan (76.2%), Shibei (73.6%), Laoshan (78.5%), and Pingdu (70.0%). Shinan District had the highest total number of node occurrences. Instructional time-related nodes had the lowest proportion in most districts.

Because districts differed in the number of included articles and in the number of compulsory education schools, absolute frequencies were not directly comparable. Two complementary measures were therefore calculated: the mean distinct nodes per included article and the node occurrences per school. The first captures how many different reform narratives a district’s articles mentioned on average. The second captures how frequently schools in a district were drawn into the coded reform narratives relative to the size of its school system. Both adjusted measures showed a similar pattern of uneven distribution across districts.

As noted in section 2.4, these comparisons are based exclusively on the 376 included articles, as the total number of posts published by each account could not be reliably retrieved. They therefore do not reflect overall district communication activity, and they should not be taken as evidence of actual school-level implementation.

## 4 Discussion

### 4.1 Main findings

This study identified 25 narrative types of school time reform across instructional time, recess, and after-school services, based on a directed content analysis of 376 official WeChat articles. Within this typology, physical activity emerged as a theme with high discursive visibility across all three dimensions. Focusing on this emergent theme, the discussion below examines three aspects of the findings: first, the distribution of physical activity-related types across the three dimensions and possible explanations for the differences in their frequencies, although the present data cannot determine the underlying mechanisms; second, differences in the frequency of coded nodes across districts; and third, how physical activity was described in relation to health and participation. Throughout the discussion, discursive visibility refers to article-level frequency, as defined in section 2.1.

#### 4.1.1 Physical activity as a frequently reported theme in school time reform narratives

In instructional time, PE guarantees were frequently reported. “Guaranteed PE instructional time” (30 occurrences) and “PE instructional time quality assurance” (18 occurrences) together accounted for the majority of instructional time narratives, which may reflect the influence of national policies mandating minimum daily physical activity (2, 4, 5). At the same time, this pattern may partly echo international evidence that the structure and quality of instructional time use, including time for physical activity, deserve equal attention alongside the quantity of time allocated (15–17, 20).

In recess reform, “Ground space gamification” (38 occurrences) and the revival of traditional games were reported in many articles as ways of enriching recess time and promoting active participation. The present study cannot determine whether these practices affect student physical activity. However, prior research has suggested that recess quality may affect student physical activity levels, with strategies such as providing varied play equipment and embedding PE content into recess possibly increasing moderate-to-vigorous physical activity (24, 25). In addition, the revival of traditional games may indicate that these practices were associated not only with health benefits but also with cultural dimensions (28).

In after-school services, “Curriculum-based club activities” (121 occurrences) was the node that appeared in the largest number of articles across all 25 nodes. Of the 121 articles with this node, 70 (57.9%) specifically mentioned sports or physical activity clubs. Although the present study does not assess program quality or student outcomes, prior research has suggested that well-designed after-school physical activity programs may improve cardiorespiratory fitness, muscle strength, and agility in children and adolescents (32–34), and that teacher-student interaction quality in these settings may predict psychological need satisfaction and intrinsic motivation (35).

Taken together, physical activity-related types appeared across all three dimensions, indicating that official narratives frequently associated school time reform with physical activity. This level of reporting is notable in a national context where physical education has historically received limited emphasis relative to academic subjects, although the present data cannot determine whether this reflects actual changes in emphasis.

#### 4.1.2 Selective reporting of physical activity practices

Within physical activity-related reporting, discursive visibility was not equally distributed across types. Comparatively, event-based and narrative-rich practices, such as sports clubs, ground games, and traditional games, were reported more frequently.

Before interpreting this unevenness, we consider alternative explanations. Some of the unevenness across dimensions may reflect structural factors, such as the larger number of after-school nodes and the stronger policy emphasis on after-school services. However, these factors do not explain the asymmetries within each dimension. Within instructional time, “Guaranteed PE instructional time” and “PE instructional time quality assurance” were reported more frequently than “Flexible class period scheduling” or “Weekly block reorganization”; within recess, “Ground space gamification” was reported more frequently than “Safety assurance mechanism.” Since the coding scheme did not privilege physical activity in advance, these within-dimension patterns are unlikely to be fully explained by node structure or policy emphasis alone.

The uneven reporting within physical activity-related practices may be interpreted through three possible, interrelated explanations. First, it is possible that structural changes were less often reported because they may be less common in practice. Adjusting class periods, reorganizing weekly timetables, and redesigning instructional time require substantial institutional effort; schools may therefore have had fewer such practices to report.

Second, the communicative qualities of different practices may also play a role. Within physical activity-related reporting, event-based and narrative-rich practices appeared more amenable to official social media reporting. For example, one article provided a detailed account of a school-wide rope-skipping event during recess, in which students from different grades competed in group jump and freestyle rope skipping (2–17). Another article from Licang District quoted a student who explained that sports allowed him to “show myself” “win honor for the school” and enter a preferred high school (2–2), illustrating the narrative and human-interest quality of such reports.

Third, it is also possible that district education bureaus had an institutional incentive to demonstrate reform progress through communicable achievements. One article from Shinan District reported that the district ranked first in the province in primary-level student fitness testing in 2022 and organized more than 230 sports events involving nearly 20,000 athletes in 2023 (1–11). Such examples suggest that what is reported may be shaped not only by what is readily communicable, but also by what can serve as evidence of administrative performance.

The observed pattern is consistent with the possibility that official social media may selectively report certain narratives more frequently (41, 42), although the present data do not allow a direct test of the underlying mechanisms. In addition, this interpretation may be understood in relation to emerging research that treats social media content as a form of institutional discourse through which government agencies construct and communicate policy images (38, 39).

#### 4.1.3 Uneven discursive visibility across districts

As reported in section 3.6, the discursive visibility varied across districts, even after adjusting for article and school counts. These differences may partly reflect the scale of activities available to report, or differences in communication capacity; the present data cannot distinguish between these possibilities. Importantly, these comparisons are based exclusively on the sampled articles and do not reflect overall district communication activity or actual implementation.

Because physical activity-related types were among the most frequently reported across the corpus, the uneven distribution of nodes across districts suggests that the frequency with which physical activity-related practices were reported may also have varied across districts. This pattern connects with equity concerns under the double reduction policy, which aimed to reduce reliance on private tutoring and to support students through school-based learning and activities (36). Research has cautioned that schools in low-income communities face disadvantages in accessing quality resources (37), and that the policy may widen disparities when provision depends on local resources (30, 31). The present study adds a discursive dimension to this concern: if physical activity-related practices in certain districts were less frequently reported, they may have been less present in policy attention. However, the data cannot determine whether this reflects actual provision or communication capacity. The study focuses on discursive patterns in official communication; lower node frequency does not imply lower implementation quality.

#### 4.1.4 Physical activity framing in health, participation, and examination contexts

The frequent reporting of physical activity also raises questions about how official communication framed the expansion of physical activity time. In China, physical education at the junior secondary level is situated within an examination context. Since 2020, national policies have mandated that PE be included as a scored subject in the high school entrance examination, with scores to be progressively increased (45). These institutional arrangements suggest that the expansion of PE time at the secondary level may be shaped not only by health-promotion goals but also by the examination context. Studies have noted that such examination-driven systems may narrow the scope of physical education toward measurable fitness outcomes, a tendency sometimes described as “examination-driven PE” in teaching and learning practices (46). Others have pointed out that the competitive and selective nature of the high school entrance examination may raise equity concerns, particularly when it functions more as a ranking-selective examination than as a feedback-diagnostic one (47). In this context, physical activity practices are not homogeneous in their inclusiveness: some are competitive and selective, such as sports clubs oriented toward competition or examination-related training, while others are participatory and inclusive, such as ground games and traditional games. This distinction provides a useful backdrop for interpreting the discursive patterns observed in this study.

Within the sampled articles, however, such tensions rarely appeared. Only a few of the 376 articles explicitly mentioned the high school entrance examination, and most framed physical activity in terms of participation, enjoyment, and health. Examination-driven objectives tended to appear in indirect language, such as integrating Grade 9 PE test items into recess activities, and the high school entrance examination was often replaced by expressions like “strengthening school quality” or “physical fitness and health.” Official reporting thus tended to present different forms of physical activity under a broad language of participation, enjoyment, and holistic development. For example, one PE demonstration class report concluded that the school would help “every student” “enjoy fun, strengthen physique, improve personality, and temper will in physical exercise” (2–3). In these articles, competitive and selective dimensions of PE were rarely described, while the vocabulary of holistic development was more frequently used.

### 4.2 Implications for school-based health promotion

One possible implication of these findings is that official education media frequently reported PE guarantees, recess activities, and after-school sports clubs when narrating school time reform. Official narratives appeared to present these changes not merely as adding minutes, but as improving the quality of time use to develop physical activity. This pattern may suggest that, in official communication, school time reform was often associated with physical activity. This finding might be tentatively related to the WHO’s recognition that schools are critical settings for health promotion (48), and that the organization of school time can shape opportunities for physical activity (49). However, this study focuses on discursive representation rather than actual impact.

A further possible implication concerns the differences in reporting frequency, which may raise considerations for the public communication of school health practices. Event-based and narrative-rich practices were more frequently reported in the sampled official narratives. This pattern may suggest that the frequency with which school health practices are reported could be associated with how readily they can be presented as compelling stories, although the present data cannot determine the relative importance of different practices.

### 4.3 Broader contributions

First, this study provides a worked example of how school time reform can be analyzed through a three-dimensional framework integrating instructional time, recess, and after-school services. This dual-axis analytical strategy, capturing both time quantity and time quality, may offer a replicable approach for policy content analysis in other contexts.

Second, the inductively developed typology of 25 narrative types provides a conceptual map of how school time reform is discursively constructed at the grassroots level, and may serve as an empirically grounded coding instrument for future comparative research.

Third, this study illustrates the potential of official social media data for examining the public communication of school-based health practices. Such data can be used to examine which reform types are reported, how frequently they appear, and how their frequency is distributed across districts, rather than serving only as a record of policy implementation.

### 4.4 Research limitations and future directions

Several limitations should be noted.

First, this study is limited to discursive representation in official media narratives and does not examine actual implementation or effectiveness. The frequency counts are based on the 376 included articles, and district comparisons therefore reflect the sampled articles rather than overall district communication activity. Future research could triangulate these findings with school administrator interviews, teacher surveys, and direct observation to examine implementation and test possible explanations.

Second, the initial screening of candidate articles was conducted by one author. Although inclusion and exclusion criteria were defined in advance and applied consistently, the absence of independent double screening may have introduced selection bias. Future studies could incorporate independent dual screening to strengthen the transparency of corpus construction.

Third, the analysis was limited to textual content and to the article-level presence of each type. It did not examine images or image–text relations, nor did it capture the depth or detail of reporting: a brief mention and an extensive report were treated equivalently. Future research could incorporate image-level analysis and measures of reporting depth to examine these aspects further.

Fourth, this study did not establish links between specific reforms and student health outcomes. Future research could employ quasi-experimental or longitudinal designs to assess the impact of school time reform on student physical activity levels, fitness, or well-being.

Finally, the study focused exclusively on the supply side of reform discourse. Future research could examine how different audiences interpret and respond to these official narratives about school health practices.

## Data Availability

The original contributions presented in the study are included in the article/Supplementary material, further inquiries can be directed to the corresponding author.
