# Supplementary material for: Discursive visibility of physical activity in school time reform: an analysis of district education bureaus’ official media narratives
Source: Front Public Health. 2026 Sep 30;14:1919160. doi: 10.3389/fpubh.2026.1919160 (PMC13662100; doi:10.3389/fpubh.2026.1919160)
Supplement: Supplementary file 1 [file Table_1.DOCX]

# **Supplementary Material 1: Search Terms for WeChat Article Retrieval**

| **Initial search terms** | | **Supplementary Search Terms** |
| --- | --- | --- |
| 课时  （Instructional time） | 课时要求  （Instructional time mandatory norms） | 时间（Time）  课程表（Class schedule）  课堂（Class hours）  课程（Curriculum schedule）  弹性课时（Flexible scheduling）  长短课（Long and short class periods）  大单元（Large-unit teaching）  课时监管（Teaching hour monitoring）  时间调控（Monitoring of class hours）  15分钟大课间（15-minute recess）  休息（Break）  延长休息（Extended break）  游戏（Games）  运动（Exercises）  延长课间（Extended recess）  阳光大课间（Sunshine recess）  延长课后（Extended after-school）  晚自习（Evening self-study）  初中晚自习（Junior secondary evening self-study）  托管（Specialty care）  作业（Homework） |
|  | 课时结构  （Instructional time structural optimization） |  |
|  | 课时管理  （Instructional time implementation monitoring） |  |
| 课间  （Recess） | 课间时长调整  （Recess duration adjustment） |  |
|  | 课间活动设置  （Recess activity enrichment） |  |
|  | 课间调控管理  （Recess organization and management） |  |
| 课后  （After-school services） | 课后时间结构  （After-school service time structure） |  |
|  | 课后内容项目  （After-school service content and programming） |  |
|  | 课后管理机制  （After-school service management mechanisms） |  |

**Note:** Initial search terms were derived from the first-level and second-level nodes of the coding framework. Supplementary terms emerged during iterative reading and were not assigned to specific nodes, as they served only to broaden the retrieval net. Search terms served only to generate a candidate pool; final inclusion or exclusion was determined through manual reading against the three inclusion criteria (see Section 2.3). Coding was conducted on the original Chinese texts.
