# Supplementary material for: Discursive visibility of physical activity in school time reform: an analysis of district education bureaus’ official media narratives
Source: Front Public Health. 2026 Sep 30;14:1919160. doi: 10.3389/fpubh.2026.1919160 (PMC13662100; doi:10.3389/fpubh.2026.1919160)
Supplement: Supplementary file 2 [file Table_2.DOCX]

# Supplementary Material 2：Codebook for School Time Reform Narratives

| **First-Level Node** | **Second-Level Node** | **Third-Level Node** | **Node code** | **Definition** | **Example Quotes (Source ID)** |
| --- | --- | --- | --- | --- | --- |
| **Instructional Time** | Instructional time mandatory norms | Guaranteed PE instructional time | I-1 | Refers to official WeChat public account articles reporting that schools have implemented or increased the number of PE class hours per week to meet national or local policy requirements for daily physical activity. | All primary and secondary schools in Licang District actively implement the Opinions on Implementing the Student Physical Fitness Enhancement Plan, taking measures such as ... adhering to one PE class per day ... to ensure that students engage in at least 2 hours of comprehensive physical activity daily (2-3) |
| **Instructional Time** | Instructional time mandatory norms | Guaranteed labor education instructional time | I-2 | Refers to official WeChat public account articles reporting that schools have incorporated labor education into the formal curriculum and ensured class hours meet requirements. | At the same time, the school has appointed full-time labor education teachers and, on the basis of fully offering, adequately scheduling, and effectively teaching one labor education class per week, ensures at least 30 minutes of labor time every day (1-52) |
| **Instructional Time** | Instructional time mandatory norms | Guaranteed aesthetic education instructional time | I-3 | Refers to official WeChat public account articles reporting that schools have ensured class hours for aesthetic education (music, art, calligraphy, etc.). | Shibei District’s school aesthetic education work builds a curriculum system, a practice system, and an evaluation system, effectively improving the quality and level of school aesthetic education (3-5) |
| **Instructional Time** | Instructional time structural optimization | Flexible class period scheduling | I-4 | Refers to official WeChat public account articles reporting that schools adopt flexible class periods. | Each class period was adjusted to 35 minutes, with 2.5 hours of outdoor physical activity daily, and 10-minute short sessions scheduled in the morning and afternoon for campus routine tasks (1-17) |
| **Instructional Time** | Instructional time structural optimization | Weekly block reorganization | I-5 | Refers to official WeChat public account articles reporting that schools reorganize class hours on a weekly basis, such as thematic weeks or integrated activity blocks. | Schools shall provide after-school services on all five weekdays, for at least two class periods per day, and the ending time shall be aligned with the normal off-duty time in each district and city (6-2) |
| **Instructional Time** | Instructional time implementation monitoring | Supervision and inspection arrangements | I-6 | Refers to official WeChat public account articles reporting on monitoring, inspection, or supervision activities conducted by education authorities or schools regarding instructional time implementation. | The school established a task force for implementing classroom teaching basic standards, headed by the principal, strictly implementing the weekly classroom teaching research and discussion model (5-3) |
| **Instructional Time** | Instructional time implementation monitoring | PE instructional time quality assurance | I-7 | Refers to official WeChat public account articles reporting on efforts to ensure PE lesson quality through teaching research, teacher training, and innovative curriculum design, etc. | Teachers designed the technical learning as an engaging level-breaking game: Level 1 Tuck and Roll Fun ... Level 2 Support Master ... Level 3 Perfect Move Show ... (2-4) |
| **Recess** | Recess duration adjustment | Recess time extension | R-1 | Refers to official WeChat public account articles reporting that schools have extended the standard recess from 10 to 15 minutes or longer. | Since piloting the recess reform in 2023, Qingdao Chongli Primary School has embraced the health-first philosophy, turning the 15-minute recess into a vitality station for students’ physical fitness improvement through scientific design and tiered activities (2-35) |
| **Recess** | Recess duration adjustment | Diversified recess time configurations | R-2 | Refers to official WeChat public account articles reporting that schools have designed multiple recess periods (e.g., morning and afternoon long breaks, grade-differentiated schedules). | The school innovatively creates a Sunshine, Joyful dual extended recess model, ensuring students engage in no less than 2 hours of daily physical activity (7-11) |
| **Recess** | Recess activity enrichment | Ground space gamification | R-3 | Refers to official WeChat public account articles reporting that schools have painted game patterns (hopscotch, number grids, etc.) on playground surfaces to encourage active play. | Innovating the Game+ model, such as Garbage Sorting Hopscotch ... (10-1) |
| **Recess** | Recess activity enrichment | Educational indoor activities | R-4 | Refers to official WeChat public account articles reporting on indoor recess activities such as desk exercises, hand gesture dances, or indoor games. | Tonghe Primary School launched dynamic recess hand-gesture dance activities. As the music changed, pairs of little hands traced graceful arcs in the air (5-11) |
| **Recess** | Recess activity enrichment | Ball-game recess activities | R-5 | Refers to official WeChat public account articles reporting on ball games during recess, including basketball, football, volleyball, table tennis, etc. | Qingdao No. 51 Middle School’s Principal’s Cup football match, themed Youth Passion, Friendship Together (1-51) |
| **Recess** | Recess activity enrichment | Interdisciplinary extension of recess activities | R-6 | Refers to official WeChat public account articles reporting that recess activities integrate content from multiple subjects (e.g., Chinese, math, science) for holistic education. | Jinggangshan Road Primary School launched a Searching for Spring interdisciplinary extended recess adventure ... immersing in the wonderful fun brought by multidisciplinary integration (4-1) |
| **Recess** | Recess organization and management | Phased promotion mechanism | R-7 | Refers to official WeChat public account articles reporting on step-by-step implementation plans for recess reform. | Schools in Licang District, in light of their own characteristics and venue conditions, scientifically design colorful extended recess activity plans (2-1) |
| **Recess** | Recess organization and management | Safety assurance mechanism | R-8 | Refers to official WeChat public account articles reporting on safety measures, teacher supervision, or emergency protocols for recess. | Teachers place special emphasis on safety education, ensuring that every student masters proper protection and assistance methods through the Little Guardian of Protection training (2-4) |
| **After-School Services** | After-school service time structure | Segmented activity time model | A-1 | Refers to official WeChat public account articles reporting that after-school time is divided into functional segments (e.g., homework tutoring, club activities). | Kutao Primary School implements a Three-Segment service model: homework tutoring + after-school physical activities + specialty services. A 30-minute extended recess physical activity and class cultural and sports activities are arranged every afternoon (6-27) |
| **After-School Services** | After-school service time structure | Flexible after-school service boundaries | A-2 | Refers to official WeChat public account articles reporting on flexible dismissal times, staggered pick-up, or extended service hours. | The school designated the first week as a trial week, allowing parents to figure out their preferred pick-up times. After the trial, the school contacted parents again to confirm service hours and days based on their trial experience (1-6) |
| **After-School Services** | After-school service content and programming | Menu-based course provision | A-3 | Refers to official WeChat public account articles reporting that schools offer a menu of courses for students to choose from based on interest. | Our school uses after-school service time to enrich the development of its distinctive culture and special courses, so that these distinctive initiatives can better serve students’ development (6-2) |
| **After-School Services** | After-school service content and programming | Curriculum-based club activities | A-4 | Refers to official WeChat public account articles reporting that schools have systematically integrated club activities into the after-school curriculum, including both sports-related and non-sports clubs. | Qingdao Zaoshan Primary School offers over 30 Curriculum-based clubs including drama, roller skating, chess, football, rugby, wind ensemble, and aerobics (2-7) |
| **After-School Services** | After-school service content and programming | After-school homework management | A-5 | Refers to official WeChat public account articles reporting on homework guidance, tiered assignments, and ensuring homework completion at school. | In the area of homework, the school is committed to a modern interpretation of “precision homework and tailored instruction.” Focusing on reducing burdens and improving quality and on integrating the five domains of education, it has built a full-chain homework system (6-12) |
| **After-School Services** | After-school service management mechanisms | After-school service supervision | A-6 | Refers to official WeChat public account articles reporting on quality monitoring, inspection, or evaluation of after-school services. | Laoshan District optimizes homework management through information technology, using the Data Cockpit of the Teaching Cloud Platform to provide regional big-data analysis, measuring the difficulty coefficient of assignments and students’ mastery levels, thereby achieving effective supervision of all schools and classes in the district (6-34) |
| **After-School Services** | After-school service management mechanisms | Parental resource integration | A-7 | Refers to official WeChat public account articles reporting that parents are recruited as volunteers to contribute to after-school programs. | The school invites parents to enter the classroom and carry out the I Come to Be the Teacher—Parent Lecturer Corps Classroom Experience activity (5-3) |
| **After-School Services** | After-school service management mechanisms | Community resource integration | A-8 | Refers to official WeChat public account articles reporting that schools integrate community resources (e.g., social organizations, experts) into after-school services. | During after-school service hours, Zhengzhou Road Primary School invited the Jiamoshan Regional Intangible Cultural Heritage—Yanjia Mountain Land Yangko from Zhoukoulu Community into the campus to carry out cultural heritage activities (3-10) |
| **After-School Services** | After-school service management mechanisms | University resource integration | A-9 | Refers to official WeChat public account articles reporting that schools partner with universities for after-school programs. | The school strengthens cooperation with universities in the district, introducing college student volunteers to participate in after-school services (1-58) |
| **After-School Services** | After-school service management mechanisms | Collaborative education mechanism | A-10 | Refers to official WeChat public account articles reporting on multi-stakeholder collaboration (school, family, community) in after-school education. | Shinan District focuses on seven major areas, group-based school management, regulating off-campus tutoring institutions, advancing after-school services, improving classroom teaching quality, integrating sports and education, sharing outstanding teachers, and collaborative education among families, schools, and society. It has introduced practical, robust, and effective measures, making efforts in multiple directions and adopting comprehensive policies to ensure that all tasks of the “double reduction” initiative are fully implemented and delivered on the ground (1-7) |
